# Unraveling enhanced brain delivery of paliperidone-loaded lipid nanoconstructs: pharmacokinetic, behavioral, biochemical, and histological aspects

**DOI:** 10.1080/10717544.2022.2069880

**Published:** 2022-05-09

**Authors:** Saleha Rehman, Bushra Nabi, Amaan Javed, Tahira Khan, Ashif Iqubal, Mohammad Javed Ansari, Sanjula Baboota, Javed Ali

**Affiliations:** a Department of Pharmaceutics, School of Pharmaceutical Education and Research, New Delhi, India; b University College of Medical Sciences, University of Delhi, Dilshad Garden, New Delhi, India; c Department of Pharmacology, School of Pharmaceutical Education and Research, New Delhi, India; d Department of Pharmaceutics, College of Pharmacy, Prince Sattam Bin Abdulaziz University, Al-Kharj, Saudi Arabia

**Keywords:** Paliperidone, schizophrenia, brain delivery, histopathological, immunohistochemical

## Abstract

Antipsychotics are accompanied by extrapyramidal side effects that deter treatment adherence and patient compliance. Paliperidone (PPD), an atypical (second-generation) antipsychotic recommended for managing schizophrenia presents biopharmaceutical challenges and pharmacological constraints which dissuade it from crossing the brain barrier. The present research aimed to assess paliperidone-loaded lipid nanoconstruct (PPD-LNC) for an improved antipsychotic activity for managing schizophrenia. High % cell viability in Neuro-2a cells (70–98%) exhibited the safety of PPD-LNC. The pharmacokinetic data showed a 3.46-fold improvement in the relative bioavailability in the brain for PPD-LNC compared to a drug suspension. The pharmacodynamic evaluation demonstrated a significant (*p* < .05) reduction in cataleptic behavior, attenuated escape latency, and prolonged stay in the open arm with PPD-LNC, thus showing its effectiveness in reducing extrapyramidal symptoms. The histopathological images further validated the safety of the formulation. Reduction in NF-κB levels as identified by immunohistochemical analysis exhibited the anti-inflammatory effect of PPD-LNC. The formulation demonstrated significant (*p* < .01) improvement in the activity of oxidative stress parameters and attenuation of neuroinflammatory markers. Based on the study findings, it was observed that formulating LNC of PPD would surmount the pharmacological constraints, improve the *in vivo* performance, and diminish the associated side effects.

## 1. Introduction

The preponderance of antipsychotics for treating schizophrenia has been endured for the past 70 years (Volkan, 2020). Both first and second-generation antipsychotics (FGAs and SGAs) have proven effective in reducing psychotic symptoms. However, SGAs or atypical antipsychotics are preferred owing to the occurrence of fewer extrapyramidal symptoms (EPS) at effective doses and improved patient-reported quality of life (Gründer et al., 2016). Generally, for a drug to cross the blood-brain barrier (BBB), it should be lipid-soluble with low-molecular-weight, which is the opposite in antipsychotics since they have limited aqueous solubility and cannot reach the target site (brain) in adequate concentrations. This leads to low bioavailability and subtherapeutic effects, and increasing their dose might result in drug-related side effects (Natarajan et al., 2017; Bellettato & Scarpa, 2018). Additionally, increasing the dose might not be a safer option due to the narrow therapeutic window. Despite advancements in the field, side effects and EPS remain a significant problem that deters treatment adherence and patient compliance (Sykes et al., 2017).

Paliperidone (PPD) is an atypical antipsychotic (second-generation) recommended for managing schizophrenia. The pharmacological action is mediated by antagonizing central serotonin type 2 (5-HT2A) and dopamine type 2 (D2) receptors (Dolder et al., 2008). Additionally, PPD exerts antioxidant and anti-inflammatory activities, which play a fundamental role in managing schizophrenia. Oxidative stress can be attributed to the enhanced dopamine activity that generates reactive oxygen species (ROS). The antioxidant defenses perceive a decline which is precedent to the neuronal damage observed in patients with schizophrenia (Caruso et al., 2020). The antioxidant activity of PPD is exerted by decreasing ROS production and reducing lipid peroxidation. In an *in vivo* study in rats, the impact of PPD (1 mg/kg for 14 days) on the redox enzymes, i.e. adenosine deaminase, catalase, and xanthine oxidase, demonstrated a significant reduction in the enzyme levels, which subsequently lowered the dopamine levels. This is important in relieving the patients of positive psychotic symptoms like delusions and hallucinations (Demirci et al., 2015). Another *in vivo* study demonstrated the potential of PPD to regulate the antioxidant and anti-inflammatory pathways in a rat model of acute and chronic restraint stress (MacDowell et al., 2016).

Further, the neuroinflammation in schizophrenia can be ascribed to the pro-inflammatory cytokines-induced microglial activation that commences the inflammatory process, which along with the neurotransmitter dysfunctions, causes neurodegeneration (Aricioglu et al., 2016). It is stated that even minor pathological alterations in the brain result in the microglia-mediated release of pro-inflammatory cytokines such as interleukin-6 (IL-6), tumor necrosis factor-alpha (TNFα), and interferon-gamma (El-Sayed El-Sisi et al., 2016). The anti-inflammatory effect of PPD is exerted by reducing the activation of microglia. Overall, PPD improves brain function by augmenting the antioxidant systems, curbing the microglial activation, and relegating the levels of pro-inflammatory mediators (Tendilla-Beltrán et al., 2021).

Although PPD holds clinical importance in the management of schizophrenia, limitations include poor aqueous solubility (0.03 mg/mL), poor bioavailability (28%), and drug evasion by P-glycoprotein (P-gp) transporters, and poor permeability dissuade it from traversing the blood-brain barrier (BBB). They are the chief reasons for its substandard therapeutic efficacy following oral administration (Rehman et al., 2021). Since schizophrenia is a dreadful neuropsychiatric disorder that warrants treatment adherence, new strategies and approaches are needed to optimize the existing therapies and reduce the associated side effects. Amongst these novel approaches, nanotechnology has been widely investigated for its potential benefits in improving drug delivery to the brain (Radaic & Martins-de-Souza, 2020).

Lately, colloidal carriers such as nanostructured lipid carriers (NLC) or lipid nanoconstruct (LNC), which are the modified form of solid lipid nanoparticles (SLN), have gained impetus in the effective management of brain disorders. LNC, after intestinal uptake, is transported via the lymphatic system to the circulatory system and then crosses BBB to reach the brain (Miao et al., 2021). Since LNC targets the drug to the active site and maintains its concentration for a sustained effect, it can effectively manage schizophrenia (Praveen et al., 2019; Mandpe & Pokharkar, 2015). In addition to this, high drug loading capacity, high entrapment efficiency, small particle size, long-term storage stability, evading hepatic metabolism, hampering P-gp-mediated drug efflux, biocompatibility, biodegradability, administration via the oral route, simple preparation method, and ease of scale-up are the well-known advantages associated with LNC (Rehman et al., 2021; Alam et al., 2015; Fatima et al., 2019). Therefore, encapsulating PPD in LNC would improve its bioavailability and penetration across BBB (Patel et al., 2016).

To address the biopharmaceutical challenges associated with PPD, formulations including solid lipid nanoparticles (SLN), microemulsion, and nanoliposomes have been prepared by the researchers. Still, these failed to achieve an effective brain delivery due to the inherent limitations. It was postulated that formulating LNC of PPD would surmount the pharmacological constraints, improve the *in vivo* performance, and diminish the associated side effects. Further, none of the previously reported studies investigated the *in vivo* effects of PPD-LNC following oral administration. Additionally, the potential of PPD-LNC in rodent’s model of ketamine-induced psychosis has also not been investigated so far. Therefore, the present research involved the assessment of PPD-LNC for these parameters. The success of the *in vivo* therapeutic efficacy of PPD-loaded LNC was determined by evaluating and comparing the bioavailability in both plasma and brain samples. The antipsychotic activity of PPD-LNC was assessed in an experimental model of ketamine-induced psychosis. Histopathological and immunohistochemical analysis was performed to determine the neuronal degeneration in the hippocampus and cortex. The antioxidant and anti-inflammatory effect of PPD was assessed using biochemical estimation of oxidative stress markers and neuroinflammatory markers. Lastly, the formulation was evaluated for its stability for three months.

## 2. Materials and methods

### 2.1. Drugs and chemicals

Paliperidone was obtained from Sun Pharmaceutical Industries Ltd. (Gurgaon, Haryana, India). Purified water was procured using Milli Q Plus (Millipore, MA, USA). Acetonitrile, water, and methanol were obtained in HPLC grades from Fischer Scientific Co. (Mumbai, India). The remaining reagents were acquired from S.D. Fine Chemicals Ltd. (Mumbai, India). Rat ELISA kits for estimation of IL-6, NF-κB, IL-10, and TNF-α were obtained from Krishgen Biosystems (Worli, Mumbai, India).

### 2.2. Animals used

Female adult albino Wistar rats weighing 150-200 g were obtained from Central Animal House, Jamia Hamdard (New Delhi, India). They were kept in polypropylene cages in a room maintained at 25 ± 2 °C/ 55 ± 5% relative humidity, with 12-h light-dark cycle and uninterrupted access to food and water. Institutional Animal Ethics Committee (IAEC) of Jamia Hamdard, registered with CPCSEA (Committee for the Purpose of Control and Supervision of Experiments on Animals) (173/GO/Re/S/ 2000/CPCSEA) permitted the study on animals (Protocol Number:1458 approved on 10^th^ April 2018).

### 2.3. Quantification of the drug via HPLC

PPD was quantified in plasma and brain samples by the method reported by Jones et al. (2009) with certain modifications. For this method, an HPLC instrument accompanied by an ultraviolet-visible detector and C18 column was used. The chromatograms were recorded and analyzed using Class VP software. The mobile phase was prepared by mixing 700 ml of 0.05 M dipotassium hydrogen orthophosphate and 300 ml of acetonitrile which was run at a flow rate of 1 mL/min. All the separations were performed at 237 nm. Stock solutions were prepared in the mobile phase, and calibration curves were drawn. HPLC method was then validated based on parameters like linearity, accuracy, precision, and robustness. The limit of quantification (LOQ) and limit of detection (LOD) for the HPLC method in plasma were 61.19 ng/ml and 121.34 ng/ml, and in the brain was 65.41 ng/ml and 198.22 ng/ml, respectively.

### 2.4. Preparation of PPD-loaded LNC

The development and evaluation of PPD-LNC have already been discussed in detail in our previous research (Rehman et al., 2021). Briefly, PPD-LNC was prepared using capmul MCM (liquid lipid), glyceryl monostearate (GMS) (solid lipid), poloxamer 407 (surfactant), and tween 20 (co-surfactant). The optimized ratio of total lipid: *S*_mix_ (surfactant-cosurfactant mixture) was 3:5, and sonication time was 11 minutes. Two methods, namely, melt emulsification-probe sonication technique (ME) and high-pressure homogenization (HPH), were employed for developing PPD-LNC, followed by quality-by-design (QbD)-based optimization. Based on the Box-Behnken design (BBD), PPD-LNC prepared using the ME method was selected as the optimized formulation since it demonstrated a small particle size of 86.35 ± 3.26 nm and high entrapment efficiency and loading capacity of 90.07 ± 1.65% and 8.49 ± 0.77%, respectively.

Assessing the optimized formulation by transmission electron microscopy (TEM) showed spherical particles with a uniform size distribution, with no indication of crystalline drug. The DSC (differential scanning calorimetry) thermograms also showed a loss of crystallinity, suggesting a molecularly dispersed state of PPD in the lipid matrix. The shifting of peaks in FTIR (Fourier transform infrared spectroscopy) spectra also revealed variation in the molecular environment, which is linked to the molecular dispersion of drugs inside the lipid matrix. Further, the optimized formulation was evaluated based on different *in vitro* parameters. The findings of the *in vitro* release study disclosed that LNC undergoes burst release, in the beginning, ensued by a sustained effect. The drug release from LNC was 5, 8, and 9-folds (*p* < .001) higher in hydrochloric acid buffer pH 1.2, phosphate buffer pH 6.8, and phosphate buffer pH 7.4, compared to the drug suspension. Stability studies in simulated gastrointestinal fluids demonstrated that formulation was stable in fasted state simulated intestinal fluid and simulated gastric fluid pH 1.2.

Further, enhanced permeation potential of LNC was established via confocal microscopy and *ex vivo* permeation study. LNC demonstrated 6-fold (*p* < .05) greater drug permeability through rat intestine than drug suspension. This might be due to the small particle size (86.35 ± 3.26 nm) since particle size below 300 nm is desirable for transport across the intestine. Additionally, permeation enhancing and P-gp inhibiting properties of the lipids, surfactant, and co-surfactant also improve intestinal permeation. Further, *in vitro* lipolysis study showed that solubilization was significantly high (*p* < .001) in the aqueous medium, implying an improved absorption in the body. Based on these findings, it was deduced that LNC holds the potential of improving the solubility and permeability of PPD, which justifies its role as a prospective carrier for delivering PPD for its improved antipsychotic activity for managing schizophrenia (Rehman et al., 2021).

### 2.5. Cell line studies

The cytotoxic activity of the prepared LNC was assessed using the Neuro-2a (brain-derived neuroblastoma) cell line to assess the safety of the developed formulation. In a 96-well plate, 1 × 10^4^ cells were plated in each well with Dulbecco’s modified Eagle’s medium (DMEM), L-glutamine (2 mmol/L), fetal bovine serum (10%), streptomycin (100 μg/mL), and penicillin (100 μg/mL). The atmospheric conditions utilized for incubating these cells were 100% relative humidity, 37 °C temperature, and 95% O_2_/5% CO_2_. Different concentrations of PPD suspension, placebo LNC, and PPD-LNC (estimated as per the *C*_max_ of the drug) were used for treating the cells, followed by treatment with 5 mg/mL 10% w/v methyl thiazole tetrazolium (MTT) and incubation at 37 °C for 4 h. Lastly, 10% w/v dimethylsulfoxide was used for the treatment to solubilize the formazan crystals. A scanning multiwall spectrophotometer measured the absorbance at 570 nm (Neupane et al., 2014). The below-mentioned formula was used to calculate the % cell viability:

$$\%\;\mathrm{cell}\;\mathrm{viability}=\;\frac{\mathrm{Absorbance}\;of\;\mathrm{treated}\;\mathrm{cells}}{\mathrm{Absorbance}\;of\;\mathrm{untreated}\;\mathrm{cells}}\times 100$$

### 2.6. Pharmacokinetic study

The rats were allocated to three groups which are: (1) control (saline) (2) drug suspension (1.2 mg/kg PPD) (3) PPD-LNC formulation (1.2 mg/kg PPD). The formulations were administered using an 18-gauge oral feeding needle. The carbon dioxide inhalation procedure was used to sacrifice the rats. Intracardiac perfusion with normal saline was done to remove the blood from the brain capillaries. Each group was split into seven time-based subgroups of 0.5, 1, 2, 4, 8, 24, and 48 h, and each subset comprised three rats. Blood and brain samples were separated at specific time points. The whole brain was isolated from each rat, rinsed with isotonic phosphate buffer pH 7.4, and thawed until analysis. For collecting the blood samples, Eppendorf microcentrifuge tubes (with ethylenediaminetetraacetic acid EDTA) were used. The plasma was obtained in the supernatant by centrifuging the tubes at 4000 rpm for 15 minutes, which was stored at −20 °C till the analysis day.

#### 2.6.1. Isolation and extraction

The plasma samples were extracted using a 500 µl plasma sample and adding 0.6 M sodium carbonate-bicarbonate buffer pH 10 (0.25 mL). To this mixture, 98:2 v/v heptane–isoamyl alcohol (8 mL) was added, and the centrifuge tube was slowly revolved on a mixer for 15 min. The heptane layer was decanted and evaporated till it dried, and the remaining residue was dispersed in the mobile phase (75 μL), 20 μL of which was analyzed via HPLC (Jones et al., 2009).

Brain samples were extracted by first defrosting the rat brain and weighing 1 g of it which is added to phosphate buffer pH 7.4 (2 mL). The samples were homogenized using a tissue homogenizer, followed by extraction with ethyl acetate (2 mL). The blend was homogenized for 4 minutes and then centrifuged at 5000 × *g* for 15 min to separate the organic layer. The extraction was replicated using ethyl acetate (2 mL). After extraction, all the organic layers were mixed and subjected to evaporation to dryness under nitrogen, followed by reconstitution of the residue with mobile phase (200 μL). The mixture was mixed using a vortex mixer and then centrifuged for 5 min at 10000 × *g*. The supernatant was filtered through a 0.45 μm nylon membrane filter and injected into the HPLC system (Le Tiec et al., 2005; Garg et al., 2019).

### 2.7. Pharmacodynamic studies

The animal model of schizophrenia for pharmacodynamic evaluation of PPD-LNC was established using ketamine, a noncompetitive and nonselective N-methyl-D-aspartate (NMDA) receptor antagonist, was employed to induce symptoms similar to schizophrenia in rats. Its repeated administration to rodents imitates positive, negative, and cognitive symptoms. After an acute administration of ketamine, anomalous expression of mTOR is detected in the brain tissues linked to neurological disorders like schizophrenia (Vasconcelos et al., 2015; Xie et al., 2020).

PPD-LNC was administered to the Wistar rats via oral route for fourteen consecutive days. Ketamine (30 mg/kg) was given intraperitoneally as a single dose on the seventh day. The rats were evaluated for behavioral activity, such as the catalepsy, Morris water maze, and elevated plus-maze models. The rats were allocated to five groups (5 rats each): (1) Control (2) Ketamine (Toxic) (3) PPD-LNC *per se* (4) Ketamine + PPD suspension (5) Ketamine + PPD-LNC.

#### 2.7.1. Catalepsy study

This was performed by employing the bar test at 1, 4, 8, and 24 h in each group. For this test, each rat was carefully held by the tail, and then the forepaws were dropped gently on the bar until the rat could grasp it. The hind paws were then rested on the floor along with the tail. The stopwatch recorded the length of time for which the rats held the position. The time for which the rats had both their forelimbs on the bar was noted until 30 s. If the rats retained this position for 30 s or more, they were considered cataleptic (Muthu et al., 2016).

#### 2.7.2. Morris Water maze (MWM) test

This is an exteroceptive behavioral model to ascertain learning and memory. This model is based on swimming and involves learning to escape to a concealed platform. The study exposes the rats to four repeated training trials per day, with each trial separated at a gap of 5 min. The experiment involved a large circular pool (diameter:150 cm; height: 45 cm) filled with water (till 30 cm) and temperature regulated at 28 ± 1 °C. The pool was partitioned into four equal quadrants by fixing two threads at a right angle on the pool rim. A white platform (10 cm^2^) was dipped 1 cm underneath the water in the target pool quadrant. The rats were dropped in the pool, and for each trial, the drop location was changed. They were allowed to trace the concealed submerged platform for 120 seconds and then remain on it for 20 seconds. If the rats were not able to locate the platform, they were escorted toward the platform and then allowed to stay for 20 seconds. The time to discover the concealed platform was noted as the mean escape latency time (ELT) (Nabi et al., 2020).

#### 2.7.3. Elevated plus-maze model

This study ascertains the fear and anxiety disturbance observed in schizophrenia. The study involved a cross-shaped maze made of black plexiglass, which included two open arms (30 × 5 cm) and two closed arms (30 × 5 × 20 cm) with a central platform (5 × 5 cm) and is placed 40 cm high above the ground. The open arms represent the insecure sites stimulating the anxiety-related behavior. The anxiolytics act by decreasing anxiety and thus increasing the open arm entries of exploration time. The number of entries and the time spent in each open and closed arm were noted. The apparatus was cleaned using 70% alcohol after the completion of each trial (Pınar et al., 2015).

### 2.8. Histopathological and immunohistochemical analysis

The brain of the rats administered with a drug suspension and PPD-LNC were isolated and fixed in 10% formalin (neutral buffered), followed by slicing and embedding in paraffin wax. The slide microtone was utilized to cut transverse slices of thickness 5 μm, then stained using hematoxylin and eosin (H & E) dye. The percentage of degenerated neurons was estimated by counting the pyknotic and necrotic neurons present in brain tissues.

For immunohistochemical analysis, sections of 5 μm thickness were cut, and paraffin was removed using xylene. The brain sections were rehydrated with graded ethanol series, washed with double distilled water, exposed to antigen retrieval using citrate buffer (pH 6), and lastly allowed to cool at room temperature for 10 minutes. The background staining was removed by incubating the sections in 4% hydrogen peroxide for 15 minutes. Sections were washed thrice using tris-buffered saline and incubated with primary Nrf-2 antibody (1:100) at 4 °C overnight. After that, sections were rinsed using the buffer and then incubated for 1 h with a peroxidase-conjugated secondary antibody. The reaction was visualized using diaminobenzidine solution. Fluorescence motic microscope (Motic AE31) facilitated with Fiji software was used to observe histological alterations in brain sections and for the semi-quantification of protein expression using the reciprocal intensity method. For these immunohistochemically stained hippocampi and frontal cortex slides, the percentage of positive neurons was estimated (Iqubal et al., 2019; Sharma et al., 2018).

### 2.9. Biochemical estimation

As discussed by Wesołowska et al. (2020), since PPD exhibits antioxidant and anti-inflammatory effects, biochemical estimation of various antioxidant and neuroinflammatory markers was performed.

#### 2.9.1. Biochemical estimation of oxidative stress markers

##### 2.9.1.1. Estimation of reduced glutathione (GSH)

This was assessed using the substrate DTNB [5,5′-dithiobis-(2-nitrobenzoic acid)], –SH group which interacts with the thiol group (of GSH) to yield thionitrobenzoic acid. The final compound produces an intense yellow color, used for ascertaining the –SH groups at 412 nm. For this experiment, 200 mg of brain homogenate was mixed with 0.02 M EDTA (2 mL), 50% trichloroacetic acid (TCA) (0.4 mL), and distilled water (1.6 mL). The mixture is shaken for 15 min and centrifuged at 3000 rpm for 15 min. The supernatant obtained (2 mL) was added to DTNB solution (0.1 mL) and 0.4 M Tris buffer pH 8.9 (4 mL) to yield a yellow-colored complex, whose absorbance was noted at 412 nm. The amount of reduced glutathione was expressed as μg of GSH/mg protein (Nabi et al., 2020).

##### 2.9.1.2. Estimation of Superoxide Dismutase (SOD)

The activity SOD enzyme was ascertained by applying the approach of Misra and Fridovich (1972). The basis of the estimation is that at alkaline pH, SOD inhibits the autoxidation of epinephrine. Brain homogenate (10 μL) was added to EDTA − 0.05 M sodium carbonate buffer pH 10.2 (970 μL). The reaction was commenced by adding 20 μL of 30 mM epinephrine. SOD activity was indirectly assessed by adrenochrome, an oxidized product of epinephrine at 480 nm using 4020 M ^− 1 ^cm ^− 1^ as the molar extinction coefficient. SOD activity was expressed as units/mg of protein (Gueroui & Kechrid, 2016; Mandpe et al., 2013).

##### 2.9.1.3. Estimation of Catalase (CAT)

The catalase activity is measured as per the previously reported method (Sinha, 1972), according to which heating dichromate in acetic acid in the presence of hydrogen peroxide (H_2_O_2_) reduces it to chromic acetate. During this reaction, perchromic acid is formed as an unstable intermediate. The declining absorbance can detect the decomposition of H_2_O_2_ at 240 nm. The catalase activity is measured as the change in absorbance per unit time and is computed as nmol H_2_O_2_/min/mg protein (Mandpe et al., 2013).

##### 2.9.1.4. Estimation of Thiobarbituric acid-reactive substances (TBARS)

The malondialdehyde (MDA) level indicated lipid peroxidation and was determined as per the process reported by Ohkawa and associated with minor variation (Ohkawa et al., 1979). A blend comprising 8.1% sodium lauryl sulfate (0.2 mL), 20% acetic acid pH 3.5 (1.5 mL), and 0.8% aqueous solution of thiobarbituric acid (1.5 mL) was prepared. This mixture was transferred to the brain homogenate (0.2 mL), volume made up to 4.0 mL using distilled water and then heated for 60 min at 95 °C. The mixture is allowed to cool under tap water, followed by the addition of 15:1 v/v of n-butanol: pyridine (5 mL) and distilled water (1 mL). The mixture was centrifuged, and the absorbance was recorded for the supernatant using a UV-visible spectrophotometer at 540 nm. The MDA content was evaluated as µmol/mg of protein (Nabi et al., 2020).

#### 2.9.2. Biochemical estimation of neuroinflammatory markers using ELISA

The neuroinflammatory cytokines (IL-6 and TNF-α) in the hippocampus and frontal cortex of Wistar rats were quantified using rat-specific ELISA kits (Krishgen Biosystems). The assay was performed following the process given with the specific cytokine kit. Both the standard and samples were put in the antibody pre-coated wells and allowed to bind. This was followed by incubating the wells at the mentioned conditions and then washing using wash buffer to remove the unbound sample. An enzyme-linked polyclonal antibody, specific for cytokines, was pipetted into the wells, then incubated and washed to remove the unbound antibody. In each well, substrate solution was added, which generated blue color, which after the addition of stop solution changed the color to yellow. The spectrophotometric determination of the samples was done at 450 nm. The color intensity of the sample was proportional to the quantity of IL-6 and TNF-α, which is calculated from the standard curve (Zameer et al., 2020).

### 2.10. Stability studies

The optimized formulation of PPD was kept at room temperature (25 ± 2 °C/ 60 ± 5% RH) and elevated temperature (40 ± 2 °C/ 75 ± 5% RH) for 3 months. The samples were observed for physical appearance, precipitate formation, phase separation, caking, gas formation, liquefaction, particle size, polydispersity index (PDI), entrapment efficiency, and percent drug remained at different time intervals (0, 30, 60, and 90 days).

#### 2.10.1. Stability studies as per ICH guidelines

The stability of PPD-LNC was assessed according to the ICH guidelines. The formulation in three batches was stored at 40 ± 2 °C and 75 ± 5% RH for 3 months. After 0, 30, 60, and 90 days, samples were withdrawn, diluted with methanol, and analyzed for change in drug content by HPLC (Agrawal et al., 2021).

#### 2.10.2. Accelerated stability study according to WHO for determination of shelf life

For ascertaining the shelf life of prepared LNC, accelerated stability studies were done as specified by World Health Organization (WHO). The formulation was stored at 30 ± 0.5 °C, 40 ± 0.5 °C, and 50 ± 0.5 °C for 90 days. After specified time intervals of 0, 30, 60, and 90 days, samples were withdrawn diluted with methanol, and the remaining drug content was determined using HPLC. The log % drug remaining vs time was plotted. From the slope of the graph, the degradation rate constant (K) at different temperature were assessed. Further, the log K values were plotted against the reciprocal absolute temperature, which is the Arrhenius plot. The shelf life was then computed (Abdelwahed et al., 2006; Ali et al., 2013).

### 2.11. Statistical analysis

The results were reported as mean ± SD, and experiments were done in triplicate. The pharmacokinetic parameters were estimated using pharmacokinetic software (PK Functions for Microsoft Excel, Pharsight Corporation, Mountain View, CA). Graph Pad InStatTM software (GraphPad Software Inc., San Diego, CA) analyzed the results. One-way analysis of variance (ANOVA) was applied to the results. Statistical significance was considered at *p* < .05.

## 3. Results and discussion

### 3.1. Cell line studies

**Figure 1.**
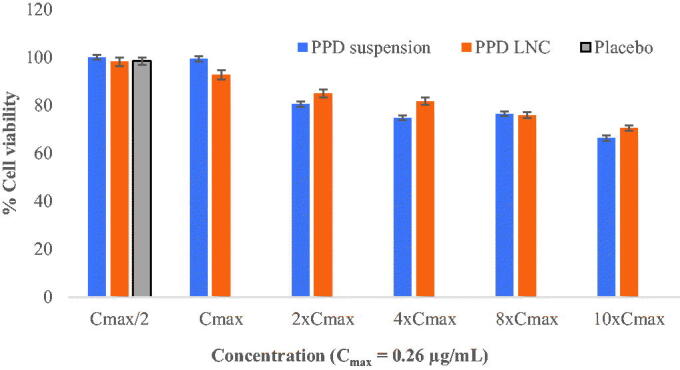
% cell viability at different levels of *C*_max_.

The % cell viability of Neuro-2a cells in the presence of PPD suspension, placebo LNC, and PPD-LNC was studied (Figure 1). The study was done at various concentrations, including the reported *C*_max_ (0.26 µg/mL). Data showed that on increasing PPD concentration from 0.13 to 2.6 µg/mL, % cell viability for LNC and drug suspension decreased, suggesting concentration-dependent uptake of the formulation. Cell viability varied from 70% to 98% for PPD-LNC and from 66% to 100% for drug suspension at each drug concentration. The placebo exhibited a cell viability of 98.45 ± 1.46% showing no cell cytotoxicity. This confirmed that lipid excipients utilized in developing LNC or placebo did not present any toxicity and fit in the GRAS category. This study validated the safety of LNC as low cytotoxicity was observed at all levels of *C*_max_ (Khan et al., 2020).

### 3.2. Pharmacokinetic study

**Figure 2.**
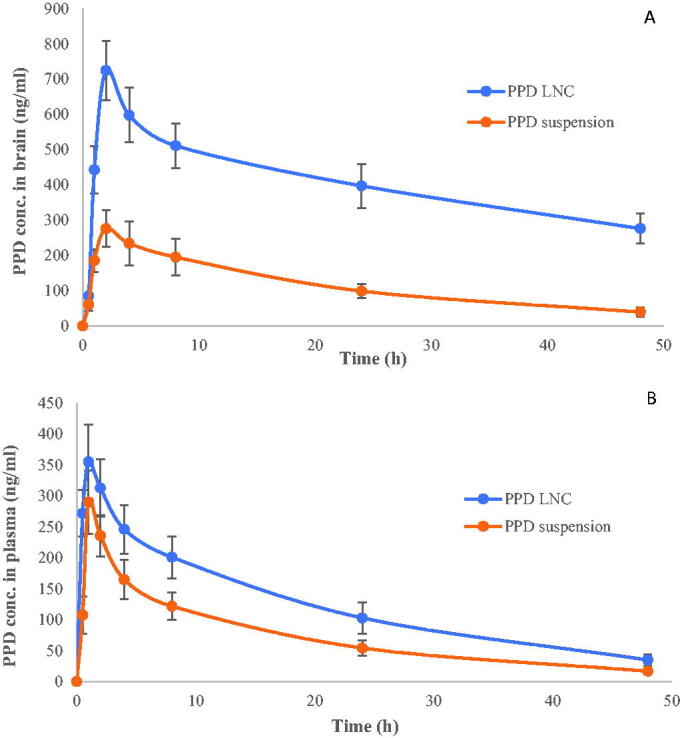
(A) Brain drug concentration and (B) Plasma drug concentration of PPD suspension and PPD-LNC.

Drug concentration in plasma and brain of Wistar rats is shown in Figure 2. The pharmacokinetic parameters are compiled in Table 1. At each time point, the brain drug concentration for the LNC group was significantly (*p* < .01) higher than drug suspension (Figure 2A). *C*_max_ in the brain for the PPD-LNC group (724.16 ± 84.28 ng/mL) was 2.63-folds greater than the drug suspension group (275.87 ± 51.93 ng/mL). However, for both the groups, *T*_max_ remained the same, i.e. 2 h. There was also a substantial increase in AUC_0-48_ of PPD-LNC (19597.11 ± 416.07 ng.h/mL) than drug suspension (5666.04 ± 81.16 ng.h/mL). The relative bioavailability for PPD-LNC was 3.46-folds greater than the drug suspension. Other pharmacokinetic parameters also showed a significant increase for LNC, thus signifying the superiority of LNC in effectively delivering the drugs to the brain. The molecular dispersion of PPD in the lipid milieu of LNC might be responsible for the improved drug concentration in the brain (Eleraky et al., 2020). The results are in harmony with the findings of Eleraky et al. where significantly superior drug concentration in the brain after oral administration of temazepam was obtained at all time points, as compared to the drug suspension (*p* < .05) (Eleraky et al., 2020). Further, higher AUC and sustained effect of PPD-NLC could be attributed to the steric stabilization effect of the surfactants, which protects NLCs from opsonization. Yu et al. also reported the reason for higher AUC and reduced elimination of miltefosine-loaded NLC as steric stabilization effect of the incorporated stearic acid, leading to the improved efficacy of miltefosine, which may result in dose reductions (Yu et al., 2021).

**Table 1.** Pharmacokinetic parameters were obtained after oral dosing of rat PPD suspension and PPD-LNC. Data expressed as mean ± SD (*n* = 3).

Pharmacokinetic parameters	Drug concentration in plasma		Drug concentration in brain	
	PPD-LNC	PPD suspension	PPD-LNC	PPD suspension
*C*_max_ (ng/mL)	354.96 ± 59.72	289.95 ± 51.25	724.16 ± 84.28	275.87 ± 51.93
*T*_max_ (h)	1	1	2	2
AUC_0-48_ (ng.h/mL)	6021.83 ± 194.03	3592.00 ± 263.75	19597.11 ± 416.07	5666.04 ± 81.16
				
AUC_0-∞_ (ng.h/mL)	6783.35 ± 147.54	3911.56 ± 4132.62	101640.50 ± 26930.99	7006.28 ± 190.35

**Figure 3.**
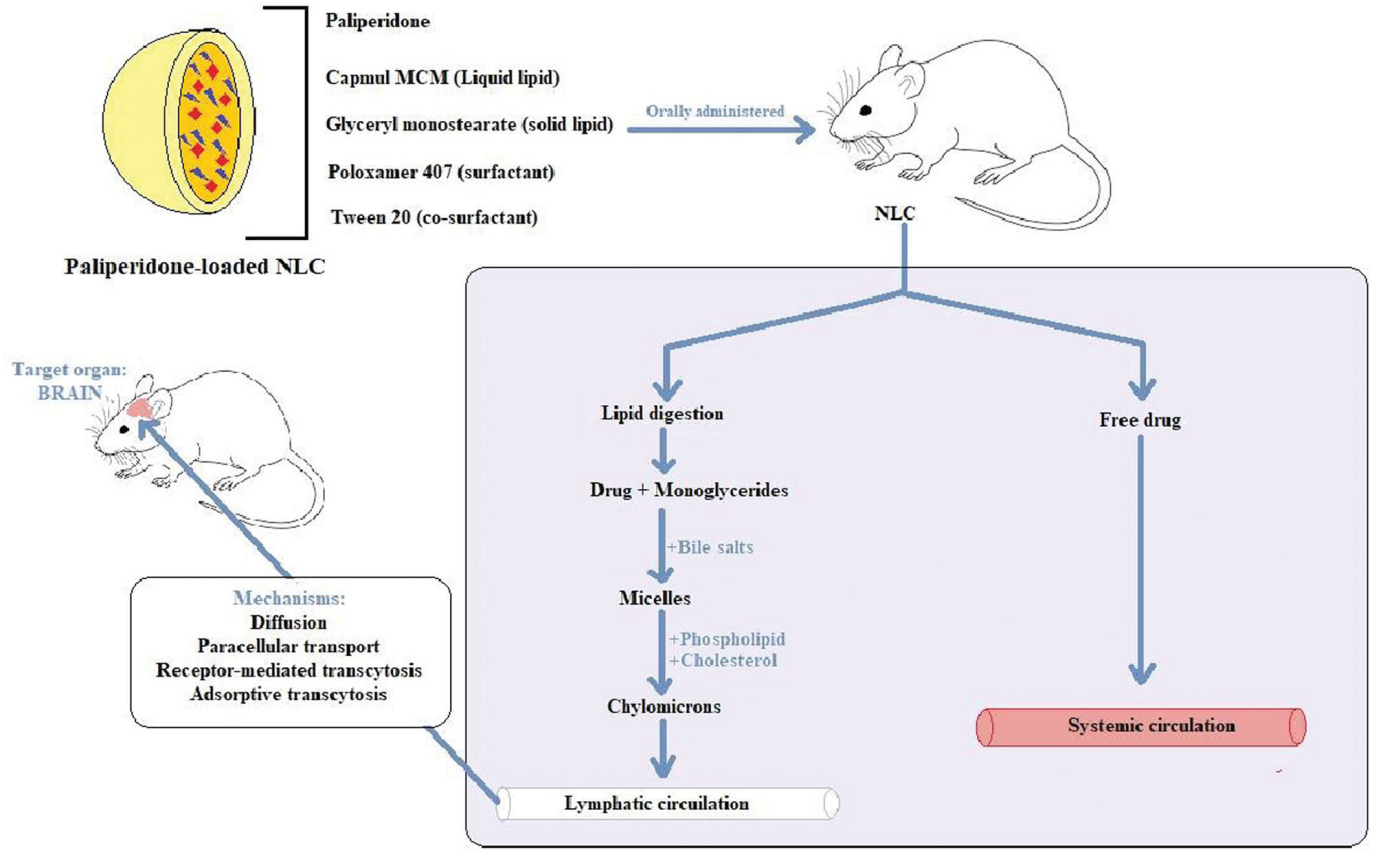
Mechanism of NLC-mediated drug delivery to the brain via the oral route.

The physiological lipids in the body simulated the fat-rich food, thus directing the bile secretion in the small intestine, which further binds with LNC to form micelles. These micelles form chylomicrons after aggregating with phospholipids and cholesterol. The chylomicrons undergo intestinal lymphatic uptake and are then transported to the circulatory system. From here, it traverses the BBB and thus reaches the target site, i.e. the brain. This mechanism has been depicted in Figure 3. Inherent characteristics of NLC when orally administered such as bypassing first-pass metabolism and lymphatic absorption were also reported by Garg et al. as the chief reasons for the 16.5-fold increase in AUC of lopinavir as compared to the pure drug (Garg et al., 2019).

Other factors contributing to enhanced brain concentration of PPD were small particle size and large surface area, protection from degradation by enzymes, reduced reticuloendothelial system uptake, and prolonged residence time. Altogether, these factors contributed to providing targeted delivery of PPD to the brain by increasing their contact time and drug concentration at the BBB surface (Miao et al., 2021; Misra et al., 2016; Singh et al., 2018). The surfactants adsorb the lipoproteins from blood and facilitate receptor-mediated transport into the brain. The P-gp inhibition by lipids and surfactants also improves the drug transport across BBB. NLC also facilitates the process of endocytosis, transcytosis, and opening of endothelial tight junctions, thus ensuring improved drug delivery to the brain (Rana et al., 2020).

Similarly, the drug concentration in the plasma was higher for PPD-LNC than drug suspension (Figure 2B). The *C*_max_ was estimated to be 354.96 ± 59.72 ng/mL and 289.95 ± 51.25 ng/mL, respectively, for PPD-LNC and PPD suspension. No difference in *T*_max_ was observed, and it remained the same for both the groups, i.e. 1 h. The pharmacokinetic parameters like AUC_0-48_, AUC_0-∞_, and K_el_ (h ^− 1^) for PPD-LNC in plasma were also greater than the drug suspension. Higher AUC and *C*_max_ indicated the enhanced bioavailability of the drug, when administered in NLC (Khaleeq et al., 2020). The relative bioavailability for PPD-LNC was 1.67-folds greater than the drug suspension. Further, it was observed that PPD concentration in the brain for the PPD-LNC group was more than the corresponding plasma concentrations at all time points. Khan and coworkers reported a 1.35-folds increase in the brain enhancement factor for carbamazepine-loaded LNC in contrast to the carbamazepine suspension. Additionally, the brain concentration of carbamazepine in the brain was significantly greater than plasma concentrations (Khan et al., 2020).

### 3.3. Pharmacodynamic studies

#### 3.3.1. Catalepsy study

**Figure 4.**
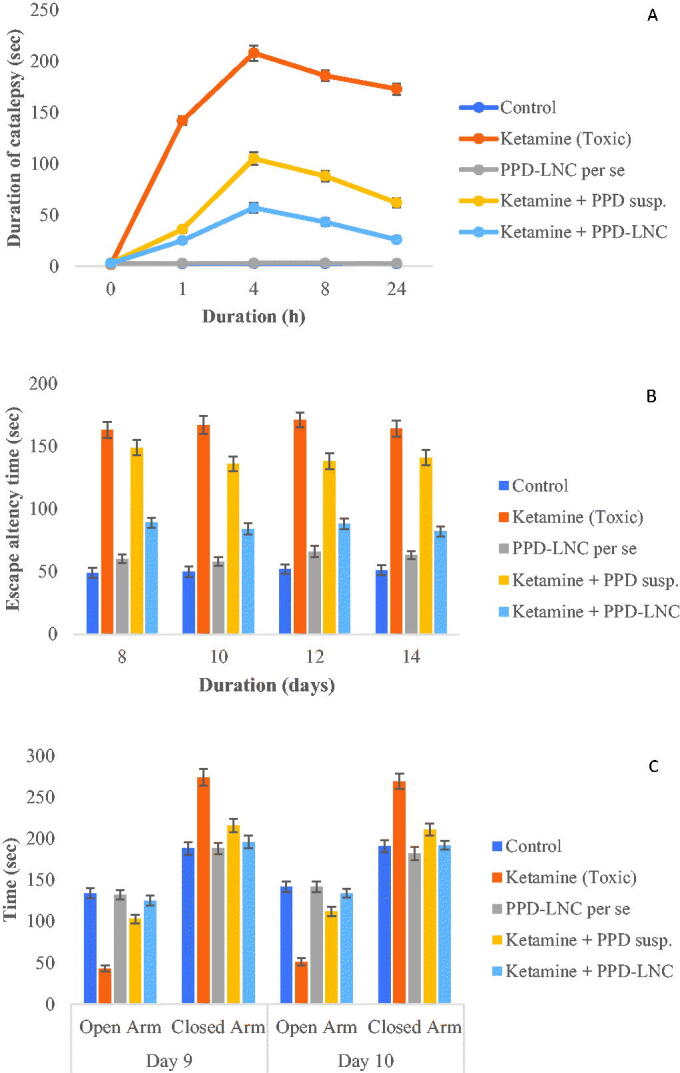
Effect of PPD-LNC on (A) Catalepsy in ketamine-induced schizophrenia in rats (B) Escape latency time in Morris water maze test (C) Time spent in open and closed arm in elevated plus maze model. Data are expressed as mean ± SD (*n* = 3).

The cataleptic effect of PPD-LNC was observed, as shown in Figure 4(A). A significant (*p* < .05) reduction in the cataleptic behavior of the rats administered with PPD-LNC was observed in comparison to the group administered with ketamine (toxic group) and the group administered with the drug suspension. PPD-LNC showed significant (*p* < .05) catalepsy at 4 h (57 ± 4.75 sec), which then underwent reduction up to 24 h. The increase in cataleptic effect up to 4 h might be due to the burst release of PPD from LNC, and the decrease thereafter can be owed to the sustained drug release from LNC. Thus, lesser fluctuation in drug concentration is observed, which contributes to prolonged antipsychotic effect and thus reduction of extrapyramidal side effects (EPS). The toxic group administered with 30 mg/kg ketamine showed the maximum catalepsy in rats. The group administered with PPD-LNC *per se* showed no antipsychotic and catalepsy activity and behaved similarly to the control saline group. The drug suspension-treated group showed a lesser reduction in cataleptic effect than the PPD-LNC treated group due to the poor absorption/release of the drug to the target site. Thus, PPD, when administered in the form of LNC, proves effective in reducing EPS. Singh et al. also reported a reduction in cataleptic response by asenapine-NLC due to the sustained drug release which leads to lesser fluctuation in drug concentration and thus provides clinical benefits of constant antipsychotic efficacy (Singh et al., 2016).

#### 3.3.2. Morris Water maze test

This test determines the effectiveness of PPD in preventing memory impairment. As depicted in Figure 4B, the escape latency time (ELT) to reach the target platform decreased gradually. The rats belonging to the toxic group (Ketamine) took the maximum time to locate the platform indicating cognitive impairment and successful induction of psychosis, thus showing maximum ELT. PPD-LNC antagonized the ketamine-induced toxicity effects and significantly (*p* < .01) attenuated the escape latency, and an improvement in learning and memory capacities was observed. PPD-LNC *per se* treated group showed similar effects as the control-treated group (Nabi et al., 2020). Vitorino et al. developed fluoxetine-loaded NLCs for the treatment of depression and examined its effects in mice using forced swimming tests. The results displayed greater anti-depressive and anxiolytic effects of NLC as compared to the drug suspension (Vitorino et al., 2020).

#### 3.3.3. Elevated plus-maze model

This study determined the efficiency of PPD in reducing the anxiety associated with schizophrenia. The rats in the PPD-LNC group demonstrated a reduction in anxiety-related behavior (Figure 4C). The rats in this group spent comparatively more time in the open arm than the closed arm. It shows that PPD acts as an anxiolytic compound, thereby increasing the open arm entries of exploration time. Contrariwise, the toxic group (administered with ketamine) showed rats in the closed arm for the maximum duration. The reduction in open arm entries represents a state of anxiety/fear in the rats administered with ketamine (Rubab et al., 2021). A study by Anand et al. also reported that rivastigmine-loaded NLC caused noticeable improvements in open arm exploration time in scopolamine-treated rats. These results were attributed to the better nasal penetration by NLCs (Anand et al., 2019).

### 3.4. Histopathological and immunohistochemical analysis

**Figure 5.**
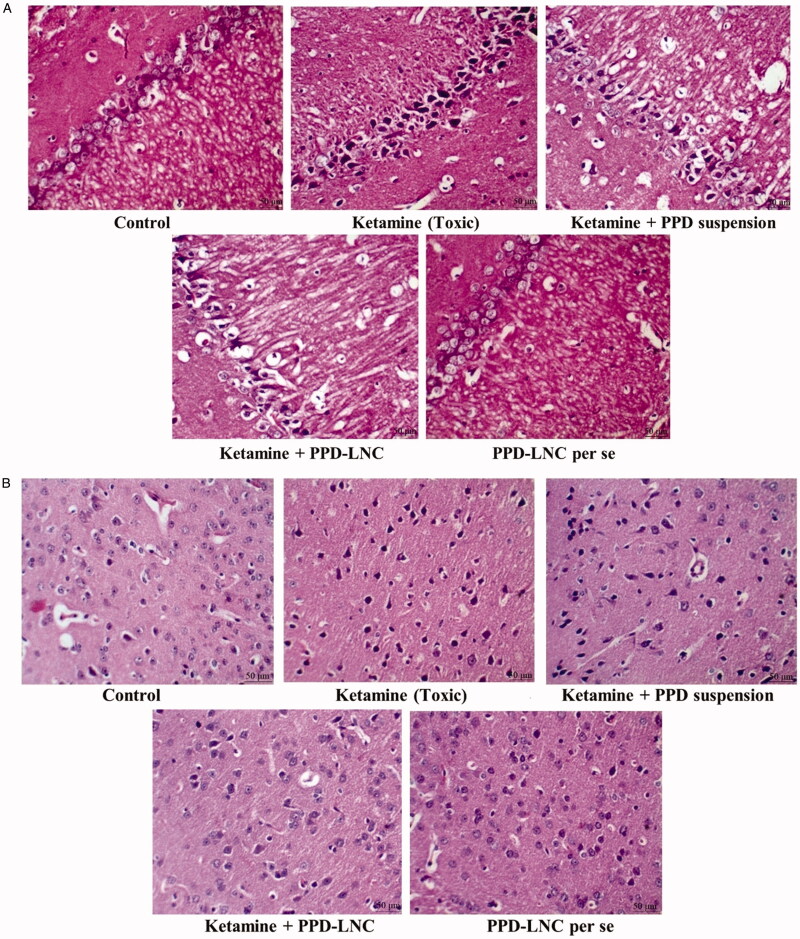
(A) H & E stained sections of the hippocampus in rats treated with normal saline (control), ketamine (toxic), ketamine + conventional formulation PPD suspension, ketamine + nanoformulation PPD-LNC, and PPD-LNC per se. (B) H & E stained sections of cortex in rats treated with normal saline (control), ketamine (toxic), ketamine + PPD suspension, ketamine + PPD-LNC, and PPD-LNC *per se*.

The histopathological study was conducted using H & E dye to stain the hippocampus and cortex region of the Wistar rats. The study examines the toxicological/structural aberrations in the brain following its administration. As shown in Figures 5A and B, the image corresponding to the toxic group (administered with ketamine) showed neurons stained in deep red color, thus confirming the toxicity. The hippocampus and cortex of the PPD-LNC-treated group showed light red color following staining with H & E. They showed a prominently visible nucleus, thus confirming the presence of healthy neurons, oligodendrocytes, and glial cells.

Additionally, there was no observation of chromatolysis, vacuolization, neuronal damage, or pyknosis in any part of the brain. As observed from the histological finding, the conventional (drug suspension) group did not exhibit any signs of toxicity. Thus, it was concluded that the prepared formulation PPD-LNC is safe for administration via oral route as no toxicity was observed. Khan et al. showed that no signs of toxicity or neuronal damage were observed in histopathological images of atazanavir-NLC. and is thus safe for oral administration (Khan et al., 2020). Additionally, Rahman et al. also revealed that histopathological images of Zerumbone-loaded NLC displayed no degenerative, vacuolar, or hemorrhagic alterations in the brain tissues (Rahman et al., 2014).

**Figure 6.**
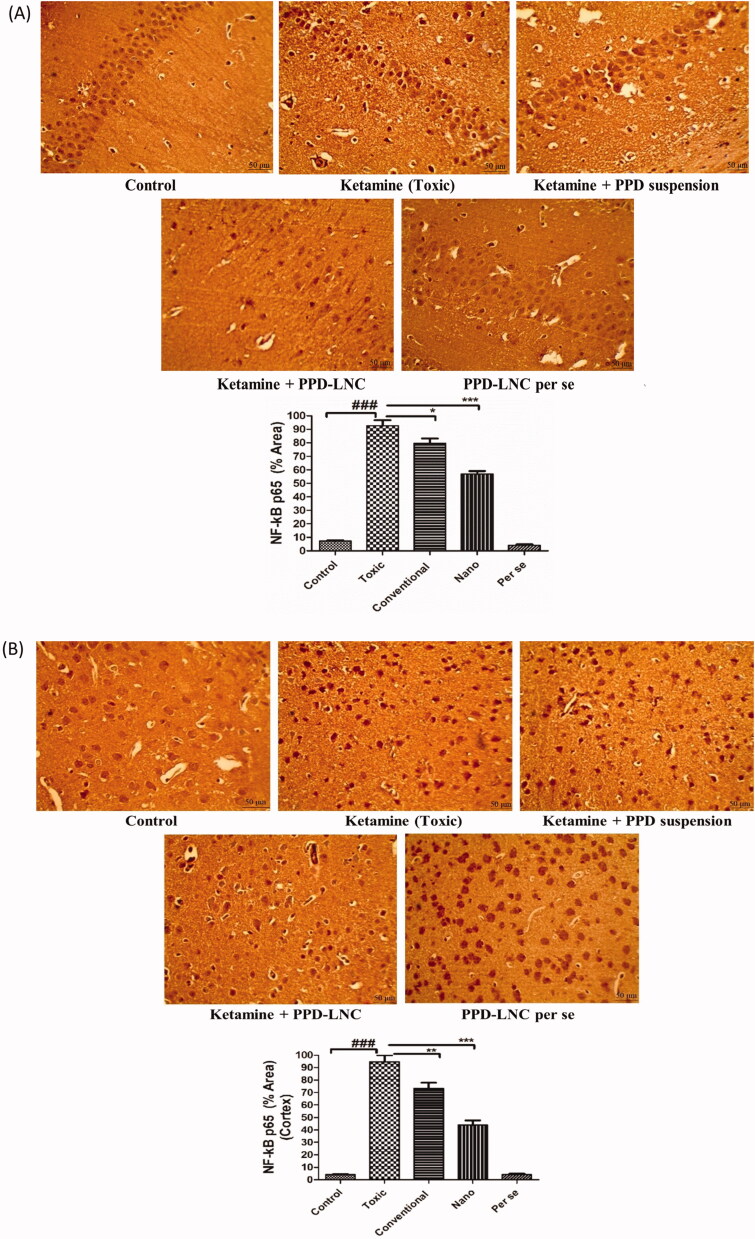
(A) Images showing the expression level of NF-κB in the hippocampus when rats were treated with normal saline (control), ketamine (toxic), ketamine + PPD suspension, ketamine + PPD-LNC, and PPD-LNC *per se*. Graphs showing the semiquantitative analysis of different treatment groups expressed as % area. The values were expressed as mean ± SEM (*n* = 6), and statistical analysis was performed using One Way ANOVA, Tukey’s multiple comparison test [scale bar- 50 μm]. (B) Images showing the expression level of NF-κB in the cortex when rats were treated with normal saline (control), ketamine (toxic), ketamine + PPD suspension, ketamine + PPD-LNC, and PPD-LNC *per se*. Graphs showing the semiquantitative analysis of different treatment groups expressed as % area. The values were expressed as mean ± SEM (*n* = 6), and statistical analysis was performed using One Way ANOVA, Tukey’s multiple comparison test [scale bar- 50 μm].

In immunohistochemical analysis (Figure 6A and B), when ketamine was administered alone, a significantly improved NF-κB expression was observed in both hippocampus and cortex when compared to the control group (*p* < .001). This might be due to the increased expression of proinflammatory mediators in the brain tissues. When ketamine was administered in combination with conventional formulation (PPD suspension), a mild reduction in the NF-κB expression was seen in the hippocampus (*p* < .05) and cortex (*p* < .01), respectively. However, when ketamine was co-administered with nanoformulation (PPD-LNC), NF-κB expression was significantly reduced in the hippocampus and cortex (*p* < .001). PPD-LNC thus successfully suppressed ketamine-induced neuroinflammation by inhibiting inflammatory markers in the brain. Rubab et al. also reported suppressed expression of *p*-NF-κB, TNF-α, and COX-2 in brain tissues from histological and immunohistochemical analysis of curcumin-loaded NLC in lipopolysaccharide (LPS)-induced depression and anxiety model (Rubab et al., 2021). Additionally, PPD-LNC *per se* was safe and exhibited a similar effect as shown in the control group.

### 3.5. Biochemical estimation of oxidative stress markers

The effect of PPD-LNC on oxidative stress parameters are presented in Table 2. In the toxic group, administration of ketamine significantly reduced (*p* < .01) the activity of endogenous antioxidant enzymes like GSH, CAT, SOD and increased the TBARS level in the hippocampus and frontal cortex compared to the control group. This leads to the accretion of oxidative free radicals producing oxidative stress and degenerative effects. The administration of PPD-LNC *per se* showed no effect on the levels of these endogenous antioxidant enzymes and behaved similarly to the control group. Conversely, PPD-LNC significantly (*p* < .01) improved the activity of GSH, CAT, SOD and reduced the activity of TBARS. Thus, PPD-LNC restored the enzyme levels to normal, indicating the antioxidant effect of PPD, which imparts neuroprotective activity (Nabi et al., 2020; Wesołowska et al., 2020; Mandpe et al., 2013). Iqbal et al. developed NLC gel of silymarin for the treatment of skin cancer. An improvement in the level of biochemical markers of oxidative stress i.e. GSH, SOD, and CAT was observed with both conventional and NLC gel, however, the antioxidant effect was more pronounced in the group treated with NLC gel (Iqbal et al., 2019).

**Table 2.** Effect of PPD-LNC on different oxidative stress markers in ketamine-induced schizophrenia in rats. Data expressed as mean ± SD (*n* = 3).

Groups	GSH μmol/mg of protein		SOD U/mg protein		Catalase nmol of H_2_O_2_/min/mg protein		TBARS nmol of MDA/mg of protein	
	Hippocampus	Frontal cortex	Hippocampus	Frontal cortex	Hippocampus	Frontal cortex	Hippocampus	Frontal cortex
Control	40.83 ± 4.12	40.93 ± 3.12	21.83 ± 2.04	22.62 ± 1.86	20.51 ± 1.83	24.13 ± 1.46	9.43 ± 1.12	9.15 ± 0.35
Ketamine (Toxic)	10.54 ± 0.94	11.38 ± 0.27	7.63 ± 0.41	7.88 ± 0.79	10.27 ± 2.47	9.24 ± 0.48	32.65 ± 3.61	31.36 ± 2.49
PPD-LNC *per se*	39.11 ± 2.89	38.73 ± 2.94	20.35 ± 1.48	21.64 ± 1.49	21.18 ± 2.15	20.98 ± 2.04	10.19 ± 0.97	9.84 ± 0.59
Ketamine + PPD suspension	19.63 ± 2.05	21.47 ± 1.71	10.23 ± 0.74	11.69 ± 0.35	14.26 ± 0.91	14.02 ± 1.25	23.81 ± 2.63	22.18 ± 1.37
Ketamine + PPD-LNC	34.86 ± 3.51	33.53 ± 2.25	16.83 ± 1.35	16.08 ± 1.14	19.23 ± 1.52	19.66 ± 1.63	27.48 ± 1.94	26.31 ± 2.61

### 3.6. Biochemical estimation of neuroinflammatory markers using ELISA

Schizophrenia is associated with neuroinflammation. The effect of the various treatment groups on neuroinflammatory cytokines in ketamine-induced schizophrenia in rats is depicted in Table 3. The results revealed the enhanced release of both the neuroinflammatory cytokines (IL-6 and TNF-α) following the administration of ketamine to the Wistar rats. However, ketamine-induced schizophrenic rats, when treated with PPD-LNC, resulted in attenuation of IL-6 and TNF-α in both hippocampus and frontal cortex, thus signifying the anti-inflammatory activity of PPD. The drug, when given alone, did not exhibit any significant variation in comparison to the control group. This anti-inflammatory effect of PPD was more evident when administered as LNC than when given as suspension since enhanced brain targeting was achieved through lymphatic uptake of LNC (Wesołowska et al., 2020). Iqbal et al. performed the biochemical estimation of TNF-α and IL-1α in mice treated with silymarin NLC gel and conventional silymarin gel, and significantly (*p* < .001) lower levels of TNF-α and IL-1α were detected in the former. Thus, it was observed that the anti-inflammatory effect of silymarin was more pronounced in NLC gel-treated mice.

**Table 3.** Effect of PPD-LNC on neuroinflammatory markers in ketamine-induced schizophrenia in rats. Data expressed as mean ± SD (*n* = 3).

Groups	TNF-α (pg/mg protein)		IL-6 (pg/mg protein)	
	Hippocampus	Frontal cortex	Hippocampus	Frontal cortex
Control	183 ± 6.32	157 ± 5.66	99 ± 2.66	94 ± 3.94
Ketamine (toxic)	432 ± 13.28	404 ± 15.39	329 ± 11.42	259 ± 11.48
PPD-LNC *per se*	179 ± 8.35	154 ± 7.46	93 ± 4.19	90 ± 4.27
Ketamine + PPD suspension	336 ± 16.29	283 ± 5.22	248 ± 8.47	207 ± 6.18
Ketamine + PPD-LNC	235 ± 10.41	212 ± 6.83	165 ± 6.93	137 ± 3.94

### 3.7. Stability studies

Based on the results, PPD-LNC was stable at 25 ± 2 °C/ 60 ± 5% RH and 40 ± 2 °C/ 75 ± 5% RH for 3 months. Visually, color and appearance were the same, and no alteration was observed. As presented in Table 4, particle size, PDI, entrapment efficiency, and % drug remained showed no change. LNC showed good redispersibility with no sediment, caking, liquefaction, gas formation, phase separation, or degradation. Small particle size and surfactant-mediated stabilization could explain its stability (Misra et al., 2016; Khan et al., 2016). According to the ICH guidelines, the formulation PPD-LNC was stored at 40 ± 2 °C and 75 ± 5% RH for three months. The shelf life of PPD in LNC as determined from the Arrhenius plot was calculated as 1.54 years at room temperature. The results thus validated that the prepared formulation was stable at different storage conditions.

**Table 4.** Stability of PPD-LNC for three months storage at 25 ± 2 °C/60 ± 5% RH and 40 ± 2 °C/75 ± 5% RH. Data expressed as mean ± SD (*n* = 3).

Period (Days)	Particle size (nm)		PDI		Entrapment efficiency (%)		% drug remained	
	25 ± 2 °C/ 60 ± 5% RH	40 ± 2 °C/ 75 ± 5% RH	25 ± 2 °C/ 60 ± 5% RH	40 ± 2 °C/ 75 ± 5% RH	25 ± 2 °C/ 60 ± 5% RH	40 ± 2 °C/ 75 ± 5% RH	25 ± 2 °C/ 60 ± 5% RH	40 ± 2 °C/ 75 ± 5% RH
0	84.1 ± 3.14	83.7 ± 2.84	0.212 ± 0.031	0.232 ± 0.029	89.8 ± 2.23	89.1 ± 1.82	99.64 ± 1.36	99.15 ± 1.22
30	85.2 ± 2.37	88.3 ± 2.54	0.246 ± 0.027	0.315 ± 0.031	89.1 ± 1.75	85.62 ± 3.12	99.43 ± 1.15	99.14 ± 1.26
60	87.1 ± 3.36	89.6 ± 2.91	0.311 ± 0.041	0.396 ± 0.053	87.7 ± 2.41	83.22 ± 2.75	98.57 ± 2.14	98.11 ± 1.32
90	87.6 ± 2.18	94.5 ± 4.76	0.329 ± 0.024	0.508 ± 0.069	86.2 ± 1.84	78.34 ± 2.19	98.32 ± 1.26	98.28 ± 1.35

## 4. Conclusions

Based on the results obtained, LNC appeared to be a potential vehicle for brain delivery of paliperidone for its improved antipsychotic activity for managing schizophrenia. The % cell viability of Neuro-2a cells validated the safety of LNC formulation as low cytotoxicity was observed at all the levels of *C*_max_. The pharmacokinetic data revealed a 3.46-fold increase in the relative bioavailability in the brain for the PPD-LNC group compared to the drug suspension. Moreover, drug concentration in the brain for the PPD-LNC group was significantly higher than the respective groups at all time points. The results of the pharmacodynamic evaluation revealed the effectiveness of PPD-LNC in reducing extrapyramidal symptoms. The histopathological images showed the absence of chromatolysis, vacuolization, pyknosis, or neuronal damage in the hippocampus and cortex of the PPD-LNC treated group. Immunohistochemical analysis showed a reduction in NF-κB levels in the hippocampus and cortex of the formulation-treated group. The biochemical estimation studies exhibited the antioxidant and anti-inflammatory activity of PPD. Finally, stability studies showed that the prepared formulation was stable at different storage conditions. Further clinical investigation of the prepared formulation is warranted to demonstrate its efficacy in the patients. A clinical benefit to the risk ratio of the developed formulation will decide its appropriateness in the management of Schizophrenia.
